# Continuous Glucose Monitoring With Linx™ Improves Glycemic Control and Satisfaction in Youth With Type 1 Diabetes: A Real-World Observational Study

**DOI:** 10.7759/cureus.91864

**Published:** 2025-09-08

**Authors:** Abeer A Abdelmaksoud, Ayman Al Hayek, Randa Matter, Nouran Salah, Hadeer H Shahin, Ali R Reyad, Nermien M Tantawy

**Affiliations:** 1 Pediatrics Department, Ain Shams University, Cairo, EGY; 2 Department of Endocrinology and Diabetes, Prince Sultan Military Medical City, Riyadh, SAU; 3 Pediatric Diabetes and Endocrinology Department, Ain Shams University, Cairo, EGY

**Keywords:** glycemia risk index, glycemic assessment, glycemic variability, patient satisfaction, type 1 diabetes

## Abstract

Background: Children and adolescents with type 1 diabetes (T1D) often have poor glycemic control and low satisfaction with management tools, increasing risks of complications. This study evaluated the clinical efficacy and user satisfaction of a novel real-time continuous glucose monitoring (CGM) system, Linx™ (Microtech Medical (Hangzhou) Co., Ltd., Zhejiang, China), over three months in adolescents with poorly controlled T1D.

Methods: In this single-center, single-arm, observational study, 75 children and adolescents using multiple daily injections were enrolled. Clinical outcomes (HbA1c, insulin dose, and hypoglycemia) and CGM metrics (time in range, glucose variability, and calculated Glycemia Risk Index) were assessed at baseline, 28, and 90 days. Patient satisfaction was measured at three months using the validated 44-item CGM Satisfaction (CGM-SAT) questionnaire.

Results: After three months of continuous Linx™ CGM use, median HbA1c significantly decreased from 9.5% to 8.1% (absolute reduction of 1.4 percentage points; p<0.001). Time in range (%TIR_70-180_) increased by 10.2 percentage points, rising from 49.8% at 28 days to 60.0% at three months (p<0.001). The frequency of hypoglycemic events dropped dramatically (p<0.001). Glucose variability improved significantly, with the coefficient of variation decreasing by 2.2%, and the composite Glycemia Risk Index declined by 11.3 points (p<0.01). Patient-reported satisfaction was high, with an overall CGM-SAT score averaging 4.8 out of 5, indicating substantial perceived benefits (mean=4.9) and low reported hassles (mean=4.6).

Conclusions: The Linx™ 15-day CGM system led to marked improvements in glycemic control and safety, along with high satisfaction, in children and adolescents with poorly controlled T1D. These results support the value of factory-calibrated CGM devices in optimizing diabetes management and quality of life in this vulnerable group.

## Introduction

Type 1 diabetes (T1D) among youth is a growing global health issue, with an estimated 1.2 million children and adolescents affected worldwide as of 2021 [1]. Adolescence is associated with a well-known decline in diabetes management, with fewer than 25% meeting recommended HbA1c targets [2]. This age group has the highest average HbA1c levels among all T1D populations [3], and glycemic outcomes are often even worse in low-resource settings [1,4]. Elevated HbA1c in adolescents is linked to higher risks of acute complications and poorer long-term quality of life, making improved glycemic control a critical priority [1,4].

Achieving reasonable diabetes control during adolescence is uniquely challenging due to a convergence of physiologic and psychosocial factors. Poor adherence to intensive management regimens is common in this age group, owing to the burdens imposed by daily insulin injections, frequent blood glucose monitoring, and dietary vigilance [1,5]. Traditional self-monitoring of blood glucose (SMBG) via fingerstick is painful and inconvenient, and truly optimizing glycemia often requires six to 10 fingerstick checks daily, which many teens struggle to sustain [6]. High diabetes distress and burnout are frequent, compounded by stigma and social pressures [1]. Even with modern devices like insulin pumps and continuous glucose monitoring (CGM), adolescents often discontinue use due to cost, discomfort, alarm fatigue, and device visibility [2]. These barriers lead to suboptimal device use and poorer glycemic outcomes, highlighting the need for teen-friendly, low-burden management strategies.

Continuous glucose monitoring (CGM) has emerged as a promising tool to address many limitations of traditional management. CGM uses a subcutaneous sensor to provide real-time glucose data and alerts, reducing the need for frequent fingersticks and easing the daily burden [7-9]. Evidence shows that CGM use in adolescents lowers HbA1c by about 0.4%, with nearly twice as many users achieving ≥0.5% reduction compared to usual care. It also increases time-in-range by approximately two hours daily and reduces extreme glucose fluctuations without increasing severe hypoglycemia [3]. Beyond glycemic improvements, CGM can positively impact quality-of-life metrics. Studies report that CGM adoption in youth is linked to reduced diabetes-related distress and improved treatment satisfaction, likely by empowering adolescents with a sense of control over their diabetes management [6].

CGM technology has rapidly advanced, improving accuracy from a mean absolute relative difference of about 25% in early devices to 9%-11% in current fifth-generation models [9]. Factory-calibrated sensors, starting with FreeStyle Libre (Abbott Diabetes Care, Inc., Alameda, CA, USA) and Dexcom G6 (Dexcom, Inc., San Diego, CA, USA), eliminated the need for fingerstick calibrations, extended sensor wear from seven to 10-14 days or more, and enabled smaller, more user-friendly devices. These innovations have increased user acceptance by reducing pain and maintenance, while offering real-time glucose data and alerts, making intensive monitoring more practical, especially for adolescents [9].

The Linx™ (Microtech Medical (Hangzhou) Co., Ltd., Zhejiang, China) CGM exemplifies recent sensor innovations, offering factory-calibrated, real-time glucose monitoring with up to 15 days of wear and an integrated sensor-transmitter design. It delivers minute-by-minute glucose data and real-time alerts via a mobile app, building on prior CGM advances by extending wear duration and maintaining zero calibration. Despite the proven benefits of CGM in improving outcomes and satisfaction, limited data exist on Linx’s clinical efficacy and user experience in adolescents with poor glycemic control. Given that higher CGM satisfaction correlates with better glucose control, assessing whether Linx™ meets clinical and user needs in this population is vital.

Accordingly, the objective of the present study was to evaluate the clinical efficacy and patient satisfaction of the Linx™ 15-day CGM over three months in children and adolescents with poorly controlled T1D. In this investigation, we specifically examine changes in glycemic metrics (including HbA1c and sensor-derived measures) and assess patient-reported satisfaction using the CGM Satisfaction (CGM-SAT) tool to determine the impact of the Linx™ CGM on both metabolic control and the user experience in a challenging T1D population.

## Materials and methods

Study design

This prospective, single-center, single-arm observational study was conducted at the Pediatrics Diabetes, Endocrinology and Metabolism Unit (PADU) of Ain Shams University Hospital (Cairo, Egypt) between January and June 2025. Participants were followed, with assessments conducted at baseline (study enrollment), 28 days, and 90 days following initiation of a CGM system. Each participant served as their own control for longitudinal comparison. The study adhered to the Strengthening the Reporting of Observational Studies in Epidemiology (STROBE) guidelines in its entirety [10] and followed the 1964 Helsinki Declaration and its subsequent amendments [11].

Participants

**Figure 1:**
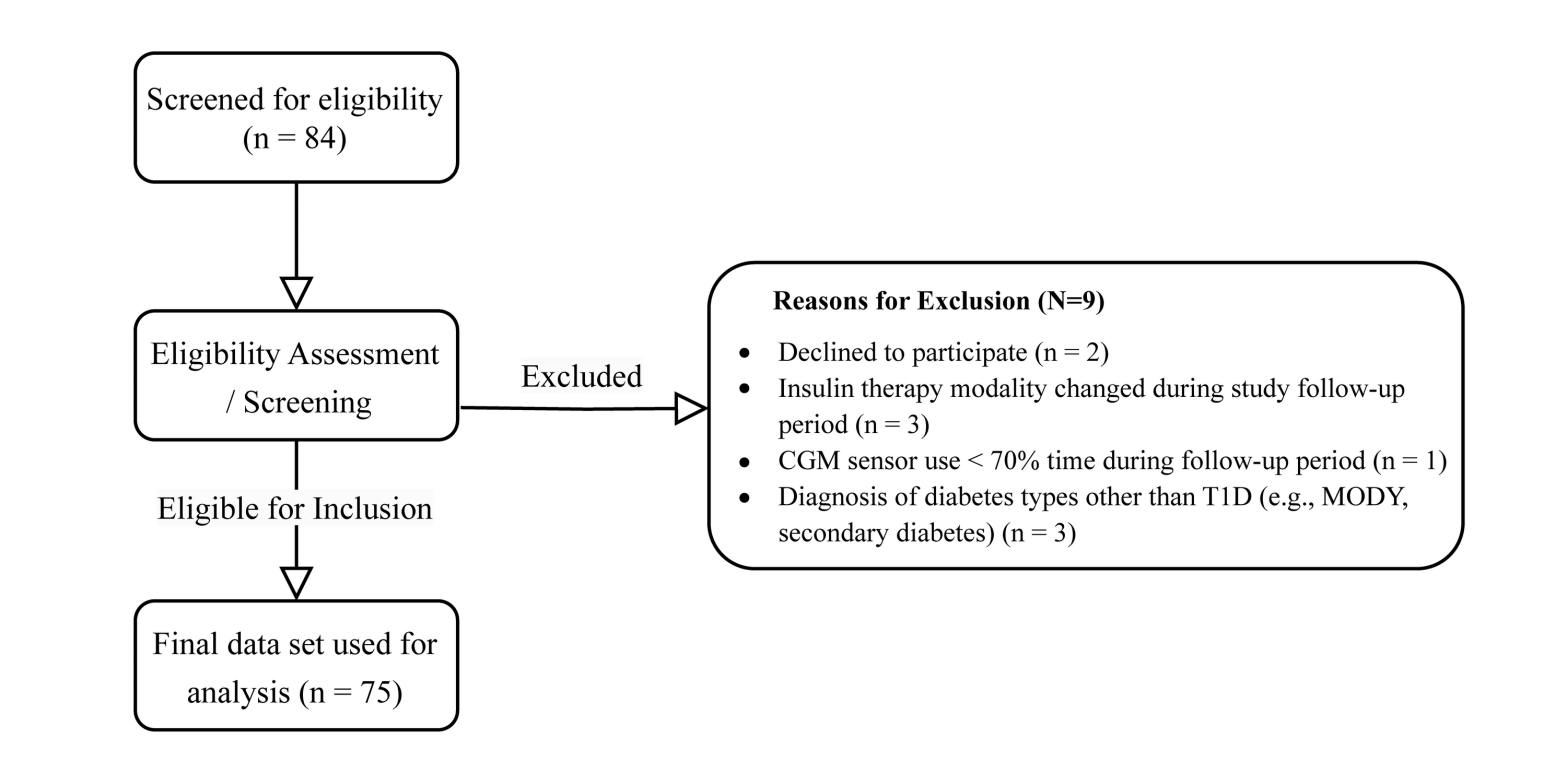
Flowchart of participant screening CGM: continuous glucose monitoring, T1D: type 1 diabetes, MODY: Maturity Onset Diabetes in the Young.

The study recruited children and adolescents aged eight to 18 years with a confirmed diagnosis of T1D, according to the International Society for Pediatric and Adolescent Diabetes (ISPAD) 2022 guidelines [12,13], with a diabetes duration of ≥1 year and after the completion of the honeymoon period. Eligible participants had been receiving subcutaneous intensive insulin therapy via multiple daily injections (MDI) for at least three months prior to enrollment and exhibited suboptimal glycemic control, defined as a glycated hemoglobin (HbA1c) A1C level of 7.5% or higher. All participants were naïve to continuous glucose monitoring (CGM), with no prior use of real-time or intermittently scanned CGM systems. All participants received routine diabetes care at the study site. Exclusion criteria were failure to achieve at least 70% CGM sensor wear time during the study period and missing data at any of the three timepoints. The 70% adherence threshold was based on international consensus guidelines recommending this minimum wear time to ensure reliable glucose profiling and metric validity [14,15]. Only those with complete sensor data at baseline, 28 days, and 90 days and who met this adherence criterion were included in the final analysis. A flowchart of participant screening is shown in Figure 1.

Intervention: Linx™ CGM system

At study entry, all participants initiated the Linx™ CGM system (Microtech Medical (Hangzhou) Co., Ltd., Zhejiang, China) [16], a factory-calibrated device with a 15-day sensor lifespan designed for placement on the back of the upper arm or the abdomen, as per manufacturer guidelines. The system allows flexible placement, with no reported difference in performance between sites. It provides real-time interstitial glucose measurements updated every minute, transmitted to a paired smartphone application. The device features customizable alarms for hypoglycemia, hyperglycemia, and rapid glucose fluctuations. Routine fingerstick calibration was not required due to factory calibration, reducing user burden and potential error. Participants received structured instruction on sensor insertion, app operation, interpretation of glucose trends, and alarm settings. Insulin therapy adjustments were not mandated by the study protocol; however, participants and their healthcare providers could make changes at their discretion using CGM data in accordance with standard diabetes care practices. Replacement sensors were provided in cases of premature detachment or malfunction, and technical support was available throughout the study period.

Outcomes

The study evaluated both clinical outcomes and CGM-derived metrics. Clinical endpoints included HbA1c (measured as %), body weight (in kilograms), total daily insulin dose (calculated as units per kilogram per day), and incidence of hypoglycemia. Hypoglycemia was defined as any sensor glucose value below 70 mg/dL or any symptomatic episode consistent with low blood glucose [12]. CGM-derived outcomes included time in range (%TIR70-180), defined as the percentage of sensor glucose readings between 70 and 180 mg/dL, as well as time in hypoglycemia (%TBR<70), time in hyperglycemia (%TAR>180), average sensor glucose, and glucose variability as measured by the coefficient of variation (%CV). The Glucose Management Indicator (GMI) was also obtained through the Pancares data management platform (MicroTech Medical Co., Ltd., Hangzhou, China). Sensor wear-time compliance, defined as the percentage of time with valid sensor data, was used to assess adherence. These metrics were computed over each wear interval and averaged across the 28-day and 90-day periods. Additionally, the Glycemia Risk Index (GRI) was calculated to quantify overall glycemic risk by integrating both hypoglycemia (CHypo) and hyperglycemia (CHyper) components [17]. CHypo was derived from the classifications of time spent in low glucose ranges: very low glycemia was defined as the percentage of time below range <54 mg/dL (%TBR<54), and low glycemia was defined as the percentage of time between 54 and 69 mg/dL (%TBR54-69). CHypo was calculated as: CHypo = [%TBR<54 + (0.8 × %TBR54-69)]. Conversely, CHyper was based on time spent in elevated glucose ranges, with high glycemia defined as %TAR181-250 and very high glycemia as %TAR>250. CHyper was calculated using the formula: CHyper = [%TAR>250 + (0.5 × %TAR181-250)]. The GRI was then computed using the weighted formula: GRI = [(3.0 × CHypo) + (1.6 × CHyper)]. Patient-reported outcomes were assessed at the 90-day visit using the CGM Satisfaction Scale (CGM-SAT), a validated 44-item instrument that evaluates user satisfaction, perceived benefits, and challenges of CGM use [18]. The CGM-SAT was administered in accordance with its original format and scoring guidelines, ensuring consistency with prior validation studies.

Data collection

Demographic and clinical data were extracted from participants’ electronic medical records at baseline and during follow-up visits on days 28 and 90. Baseline variables included age, sex, diabetes duration, HbA1c, body weight, height, and insulin regimen. Follow-up assessments included repeat HbA1c testing performed in the hospital’s central laboratory using standardized methods, along with updates on insulin dose, weight, and any reported hypoglycemic episodes.

CGM data were downloaded at each visit using the manufacturer’s software via the Pancares data management platform (MicroTech Medical Co., Ltd., Hangzhou, China). Sensor wear-time adherence was computed from the proportion of time with active glucose readings. The CGM-SAT questionnaire was completed by participants in a private setting at the final visit, with trained study staff available to provide clarification. Questionnaire responses were manually reviewed and entered into a secure research database.

Sample size and statistical analysis

The primary endpoint was the change in HbA1c from baseline to day 90. Sample size calculations were performed using G*Power version 3.1.9.7 (Heinrich Heine University Düsseldorf, Düsseldorf, Germany), targeting the detection of a clinically meaningful 0.5% reduction (two-tailed α=0.05, 95% power), assuming a within-participant SD of 1.0%, which required 54 completers [19]. Allowing for 20% attrition due to loss to follow-up, inadequate sensor wear (<70%), or incomplete data, the planned enrollment was 65 participants. In practice, enrollment exceeded this target: 75 participants met all eligibility criteria and were therefore included in the final analysis.

Statistical analyses were conducted on a complete-case basis, including only participants with available data at all three study visits. Continuous variables were assessed for normality using the Shapiro-Wilk test and visual inspection of histograms. Depending on data distribution, continuous variables are reported as mean ± standard deviation (SD) or median with interquartile range (IQR). For non-normally distributed outcomes with more than two related samples, the non-parametric Friedman test was used, followed by Durbin-Conover pairwise comparisons when significant. For outcomes with two related samples, the Wilcoxon signed-rank test was applied. All tests were two-sided, and a p-value <0.05 was considered statistically significant. Analyses were performed using SPSS version 28 (IBM Corp., Armonk, NY) and R software version 4.5.1 (R Foundation for Statistical Computing, Vienna, Austria).

Ethical consideration

The study was approved by the Research Ethics Committee of Ain Shams University Hospital, Cairo, Egypt (IRB approval No: FMASU R306/2023, 28-10-2023). Written informed consent was obtained from the parent or legal guardian of each participant, and assent was obtained from all adolescents prior to enrollment. The study was conducted in accordance with the Declaration of Helsinki and Good Clinical Practice guidelines. Confidentiality was protected through the use of anonymized data. Participants continued to receive standard diabetes care throughout the study. Study-related devices and supplies, including the Linx™ CGM system and sensors, were provided free of charge. Device-related adverse events or malfunctions were documented and reported to the ethics committee.

## Results

Clinical and metabolic characteristics at baseline

Three-month data were available for 75 participants who met all eligibility criteria and were included in the final analysis. The median age was 13.46 years, with 42 females (56%). All participants were managed with MDI. The mean duration of diabetes was 6.09 years. Median body weight was 49 kg (IQR, 37-59), and median BMI was 19.5 kg/m² (IQR, 17.8-24.8). Four participants (5.3%) reported thyroid disorders, and eight (11%) had celiac disease. Median HbA1c at baseline was 9.5% (IQR, 8.25-10.00). The median daily insulin dose was 1.10 IU/kg/day (IQR, 1.00-1.30), and the median average glucose was 200 mg/dL (IQR, 190-220). Participants experienced a median of five monthly low glucose events (IQR, 4-6). Fingerstick glucose testing frequency was as follows: 10 (13%) once daily, 26 (35%) twice daily, 21 (28%) three times daily, 12 (16%) four times daily, and 6 (8%) five or six times daily (Table 1).

**Table 1:** Baseline clinical and metabolic characteristics of study participants

Characteristic	Value
Age (years), Mean ± SD	13.46 ± 2.03
Gender, n (%)	
Males	33 (44%)
Females	42 (56%)
Treatment modality	
Multiple daily injections (MDI)	75 (100%)
Diabetes duration (years), Mean ± SD	6.09 ± 3.54
Body weight (kg), median (IQR)	49 (37, 59)
Height (cm), median (IQR)	154 (148, 161)
Body mass index, median (IQR)	19.5 (17.8, 24.8)
Comorbidities	
Thyroid disorder	4 (5.3%)
Celiac disease	8 (11%)
HbA1c (%), median (IQR)	9.50 (8.25, 10.00)
Daily insulin dose (IU/kg/day), median (IQR)	1.10 (1.00, 1.30)
Low glucose events, median (IQR)	5 (4, 6)
Average glucose (mg/dL), median (IQR)	200 (190, 220)
Daily fingerstick frequency, n (%)	
1 time/day	10 (13%)
2 times/day	26 (35%)
3 times/day	21 (28%)
4 times/day	12 (16%)
5 times/day	3 (4.0%)
6 times/day	3 (4.0%)

Comparative analysis of glycemic parameters

Glycemic control improved significantly over the study period. Median HbA1c decreased from 9.5% at baseline to 8.1% at three months (difference: -1.4%; p<0.001; Table 2). Median GMI declined from 8.7% at 28 days to 8.0% at three months (difference: -0.7%; p<0.001). Daily insulin requirements were significantly reduced. Median total daily insulin dose decreased from 1.10 IU/kg/day at baseline to 1.00 IU/kg/day at 28 days (difference: -0.10; p=0.854), further dropping to 0.70 IU/kg/day at three months (difference: -0.40 vs. baseline; p<0.001; difference: -0.30 vs. 28 days; p<0.001). Hypoglycemia burden improved markedly, with median low glucose events dropping from 5 at baseline to 0 at both follow-up time points (difference: -5; p<0.001 for both baseline comparisons; p=0.227 for both follow-up time points). Median body weight remained stable at 49 kg at baseline and 28 days, rising slightly to 50 kg at three months (difference: +1 kg; statistically significant but minimal clinical relevance).

**Table 2:** Comparative analysis of clinical and glycemic outcomes over time Data are presented as median (interquartile range, IQR), where IQR = Q1 (25th percentile) - Q3 (75th percentile). The Friedman test was used for variables with more than two related samples, with Durbin-Conover post hoc tests when significant. ^W^The Wilcoxon signed-rank test was used for two related samples. *Statistical significance indicated; P1 compares baseline vs. 28 Days, P2 compares baseline vs. 90 Days, and P3 compares 28 Days vs. 90 Days. %TIR70-180: time in range (70-180 mg/dL); %TAR181-250, time above range (181-250 mg/dL); %TAR>250, time above range (>250 mg/dL); %TBR54-69, time below range (54-69 mg/dL); %TBR<54, time below range (<54 mg/dL).

Outcome	Time Point	Value	Pairwise Comparisons
HbA1c (%)	Baseline	9.5 (8.25, 10)	p<0.001*^W^
	90 Days	8.1 (7.55, 8.5)	
Daily insulin dose (IU/kg/day)	Baseline	1.1 (1, 1.3)	P1=0.854; P2<0.001*; P3<0.001*
	28 Days	1 (0.9, 1.2)	
	90 Days	0.7 (0.6, 0.8)	
Low glucose events	Baseline	5 (3.5, 6)	P1<0.001* P2<0.001*; P3=0.227
	28 Days	0 (0, 1)	
	90 Days	0 (0, 1)	
Body weight (kg)	Baseline	49 (37, 59)	P1=0.151; P2<0.001*; P3<0.001*
	28 Days	49 (36.5, 59.5)	
	90 Days	50 (39.5, 60)	
Average glucose (mg/dL)	Baseline	200 (190, 220)	P1=0.054; P2<0.001*; P3<0.001*
	28 Days	199 (172, 224)	
	90 Days	180 (174, 185)	
Glucose Management Indicator (GMI%)	28 Days	8.7 (8.15, 9.72)	p<0.001*^W^
	90 Days	8 (7.41, 8.4)	
Sensor active percentage (%)	28 Days	98.0 (96.0, 99.0)	p<0.001*^W^
	90 Days	92.1 (88.0, 95.0)	
Glucose variability (%)	Baseline	38.2 (33.8, 40)	p=0.072^W^
	90 Days	36 (33.5, 40)	
%TIR_70-180_	Baseline	49.8 (41, 60.5)	p<0.001*^W^
	90 Days	60 (51, 63)	
%TBR_54-69_	Baseline	3 (1.5, 4.35)	p<0.001*^W^
	90 Days	1 (0, 2)	
%TBR_<54_	Baseline	1 (0.3, 2)	p<0.001*^W^
	90 Days	0.5 (0, 1)	
%TAR_181-250_	Baseline	27.6 (23.1, 31.4)	p=0.047*^W^
	90 Days	25 (23, 29.3)	
%TAR_>250_	Baseline	17 (10.8, 23.8)	p=0.197^W^
	90 Days	16 (9.8, 20.1)	
Hypoglycemia component (CHypo)	Baseline	3.2 (1.8, 5.18)	p<0.001*^W^
	90 Days	1.6 (0.8, 2.3)	
Hyperglycemia component (CHyper)	Baseline	30 (22.1, 38.5)	p=0.155^W^
	90 Days	29.3 (21.5, 33.1)	
Glycemia Risk Index (GRI)	Baseline	62.2 (48.6, 73.3)	p<0.001*^W^
	90 Days	50.9 (40.1, 58.4)	

Average glucose levels decreased. Median values declined from 200 mg/dL at baseline to 199 mg/dL at 28 days (difference: -1 mg/dL; p=0.054) and further to 180 mg/dL at three months (difference: -20 mg/dL vs. baseline; p<0.001; difference: -19 mg/dL vs. 28 days; p<0.001). Sensor wear time remained consistently high, with medians of 98.0% at 28 days and 92.1% at three months.

Time in range and glycemic exposure

At three months, median %TIR70-180 increased by 10.2 percentage points, from 49.8% at day 28 to 60.0% at day 90 (p<0.001). Time spent in level 1 hypoglycemia decreased by 2.0 percentage points, from 3.0% to 1.0% (p<0.001), and time in level 2 hypoglycemia declined by 0.5 percentage points, from 1.0% to 0.5% (p<0.001). Exposure to hyperglycemia improved. Time in level 1 hyperglycemia dropped by 2.6 percentage points, from 27.6% at day 28 to 25.0% at day 90 (p=0.047). Time in level 2 hyperglycemia decreased by 1.0 percentage point, from 17.0% to 16.0%, but this change was not statistically significant (p=0.197).

Glucose variability and Composite Glycemic Risk Scores

Glucose variability was reduced from 38.2% to 36.0% at three months (difference: -2.2%; p=0.072). The GRI improved significantly from 62.2 to 50.9 at three months (difference: -11.3; p<0.001). The CHypo was halved from 3.2 to 1.6 (difference -1.6; p<0.001), while CHyper remained unchanged (p=0.155).

Patient-reported outcomes: CGM Satisfaction

Participants reported high satisfaction with CGM, as shown in Tables 3-5 and Figure 2. The mean satisfaction score for perceived CGM benefits was 4.9 (SD=0.1; range: 4.7-5.0). The mean score for perceived hassles was slightly lower at 4.6 (SD=0.1; range: 4.2-4.8). Overall satisfaction with CGM was rated at a mean of 4.8 (SD=0.1), ranging from 4.5 to 4.9.

**Table 3:** Continuous Glucose Monitoring Satisfaction: benefits

#	Using the Continuous Glucose Monitor	Mean Score ± SD	Strongly Agree	Agree	Neutral	Disagree	Disagree Strongly
Benefits		4.9 ± 0.1					
2	Makes adjusting insulin easier	4.9 ± 0.2	71 (94.7%)	4 (5.3%)	0	0	0
3	Helps me to be sure about making diabetes decisions	4.9 ± 0.2	71 (94.7%)	4 (5.3%)	0	0	0
6	Helps to keep low blood sugars from happening	4.7 ± 0.5	51 (68.0%)	22 (29.3%)	2 (2.7%)	0	0
7	Has taught me new things about diabetes that I didn’t know before	4.9 ± 0.3	66 (88.0%)	9 (12.0%)	0	0	0
9	Teaches me how eating affects blood sugar	4.9 ± 0.3	70 (93.3%)	5 (6.7%)	0	0	0
10	Helps me to relax, knowing that unwanted changes in blood sugar will be detected quickly	5.0 ± 0.1	74 (98.7%)	1 (1.3%)	0	0	0
11	Has helped me to learn about how exercise affects blood sugar	4.8 ± 0.4	12 (16.0%)	63 (84.0%)	0	0	0
12	Helps with keeping diabetes under control on sick days	4.9 ± 0.3	68 (90.7%)	7 (9.3%)	0	0	0
17	Has helped me to learn how to treat low sugars better	4.9 ± 0.3	68 (90.7%)	7 (9.3%)	0	0	0
20	Shows patterns in blood sugars that we didn’t see before	4.9 ± 0.2	71 (94.7%)	4 (5.3%)	0	0	0
21	Helps prevent problems rather than fixing them after they’ve happened	5.0 ± 0.2	73 (97.3%)	2 (2.7%)	0	0	0
22	Allows more freedom in daily life	5.0 ± 0.0	75 (100.0%)	0	0	0	0
23	Makes it clearer how some everyday habits affect blood sugar levels	4.9 ± 0.3	67 (89.3%)	8 (10.7%)	0	0	0
24	Makes it easier to complete other diabetes self-care duties	4.9 ± 0.3	68 (90.7%)	7 (9.3%)	0	0	0
38	Has helped to adjust pre-meal insulin doses	4.9 ± 0.3	70 (93.3%)	5 (6.7%)	0	0	0
41	Has made me worry less about having low blood sugars	4.9 ± 0.3	67 (89.3%)	8 (10.7%)	0	0	0
42	If possible, I want to use this device when the research study is over	4.9 ± 0.3	67 (89.3%)	8 (10.7%)	0	0	0
43	Helps in adjusting doses of insulin needed through the night	4.9 ± 0.3	67 (89.3%)	8 (10.7%)	0	0	0
44	Makes me feel safer knowing that I will be warned about low blood sugar before it happens	4.9 ± 0.3	68 (90.7%)	7 (9.3%)	0	0	0

**Table 4:** Continuous Glucose Monitoring Satisfaction: lack of hassles

#	Using the Continuous Glucose Monitor	Mean Score ± SD	Strongly Agree	Agree	Neutral	Disagree	Disagree Strongly
Lack of hassles		4.6 ± 0.1					
4	Causes others to ask too many questions about diabetes	4.1 ± 0.7	0	0	14 (18.7%)	36 (48.0%)	25 (33.3%)
5	Makes me think about diabetes too much	4.3 ± 0.5	0	0	0	51 (68.0%)	24 (32.0%)
8	Causes too many hassles in daily life	4.9 ± 0.3	0	0	1 (1.3%)	3 (4.0%)	71 (94.7%)
14	Sometimes gives too much information to work with	4.3 ± 0.5	0	0	3 (4.0%)	47 (62.7%)	25 (33.3%)
16	Is uncomfortable or painful	4.3 ± 0.5	0	0	2 (2.7%)	50 (66.7%)	23 (30.7%)
18	Is more trouble than it is worth	5.0 ± 0.0	0	0	0	0	75 (100.0%)
25	Has caused more family arguments	4.6 ± 0.5	0	0	0	29 (38.7%)	46 (61.3%)
26	Is too hard to get it to work right	4.4 ± 0.5	0	0	0	44 (58.7%)	31 (41.3%)
27	Has been harder or more complicated than expected	4.3 ± 0.5	0	0	0	49 (65.3%)	26 (34.7%)
29	Causes our family to talk about blood sugars too much	4.2 ± 0.4	0	0	2 (2.7%)	59 (78.7%)	14 (18.7%)
30	Makes it harder for me to sleep	4.7 ± 0.7	0	3 (4.0%)	1 (1.3%)	12 (16.0%)	59 (78.7%)
31	Causes more embarrassment about feeling different from others	4.1 ± 0.4	0	0	5 (6.7%)	60 (80.0%)	10 (13.3%)
32	Shows more “glitches” and “bugs” than it should	4.3 ± 0.5	0	0	0	52 (69.3%)	23 (30.7%)
33	Interferes a lot with sports, outdoor activities, etc	5.0 ± 0.0	0	0	0	0	75 (100.0%)
34	Skips too many readings to be useful	4.7 ± 0.7	0	4 (5.3%)	0	8 (10.7%)	63 (84.0%)
35	Gives a lot of results that don’t make sense	5.0 ± 0.0	0	0	0	0	75 (100.0%)
36	Causes too many interruptions during the day	4.7 ± 0.5	0	0	3 (4.0%)	14 (18.7%)	58 (77.3%)
37	Alarms too often for no good reason	5.0 ± 0.0	0	0	0	0	75 (100.0%)
39	The feedback from the device is not easy to understand or useful	4.4 ± 0.9	0	3 (4.0%)	14 (18.7%)	10 (13.3%)	48 (64.0%)
40	I don’t recommend this for others with diabetes	5.0 ± 0.0	0	0	0	0	75 (100.0%)

**Table 5:** Continuous Glucose Monitoring Satisfaction: others

#	Using the Continuous Glucose Monitor	Mean Score ± SD	Strongly Agree	Agree	Neutral	Disagree	Disagree Strongly
1	Causes me to be more worried about controlling blood sugars	4.9 ± 0.3	0	0	0	5 (6.7%)	70 (93.3%)
13	Has shown me that blood sugar is predictable and orderly	5.0 ± 0.0	75 (100.0%)	0	0	0	0
15	Has made it easier to accept doing blood sugar tests	4.9 ± 0.2	71 (94.7%)	4 (5.3%)	0	0	0
19	Has helped my family to get along better about diabetes	4.9 ± 0.3	67 (89.3%)	8 (10.7%)	0	0	0
28	Has helped to control diabetes better even when not wearing it	5.0 ± 0.0	75 (100.0%)	0	0	0	0

**Figure 2:**
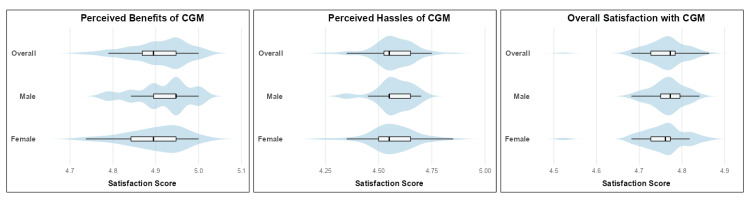
Continuous Glucose Monitoring (CGM) Satisfaction: overall score and subdomain ratings by gender

## Discussion

Our findings corroborate and extend the growing evidence that CGM substantially benefits adolescents and youth with T1D [20]. Adolescence is recognized as the period with the poorest glycemic control in T1D, with many teens failing to meet HbA1c targets in large registries [20]. In this three-month study, initiating the factory-calibrated Linx™ CGM led to significant improvements in glycemic control and high patient satisfaction. Median HbA1c declined from 9.5% to 8.1%, a substantial reduction (-1.4%) that exceeds prior trials such as the CITY study, which reported a 0.37% decrease over six months [3]. This larger effect likely reflects our cohort’s higher baseline HbA1c and greater “room for improvement,” consistent with meta-analyses showing that higher baseline HbA1c predicts larger CGM-related reductions [21].

Our participants had poor baseline control and infrequent fingerstick testing, so transitioning to CGM probably enabled more frequent, precise insulin adjustments and behavioral changes, driving the pronounced improvement. Median sensor wear was excellent at 92.1%, likely boosted by intensive education and the Linx™ device’s user-friendly features (15-day wear, no calibrations).

We observed a 10.2-percentage-point increase in %TIR to 60%, a clinically meaningful improvement that aligns with prior studies but slightly exceeds typical magnitudes, likely reflecting poorer baseline control. The CITY trial showed %TIR gains but from a better baseline (8.9% HbA1c) with a more modest %TIR70-180 [3]. Real-world registries report median %TIR of 56%-60% among youth on MDI, with only about 21% achieving the consensus >70% target [22-24]. Our findings illustrate progress and persistent gaps, indicating that full normalization of glycemia in adolescence remains challenging and may require adjunctive strategies such as intensified insulin titration, behavioral support, or hybrid closed-loop systems [22,24]. Nonetheless, the %TIR improvement is significant, as every increase correlates with lower HbA1c and substantially reduced complications [24].

Notably, these glycemic gains occurred without increased hypoglycemia. Time below range (<70 mg/dL) dropped and became within safety targets (<4%) [24], and symptomatic hypoglycemic events declined dramatically. This supports prior evidence that real-time CGM’s predictive alerts and continuous data help users prevent lows [22]. The reduction in hypoglycemia reflects timely interventions enabled by CGM alerts. We also observed reductions in glycemic variability (%CV decreased by -2.2% to 36%) and the GRI (from 62.2 to 50.9), indicating improved glucose stability. Prior studies note that greater perceived CGM benefit is associated with lower glycemic variability, consistent with our finding that high satisfaction coexisted with a flatter glucose profile [25,26].

Despite improved glycemia, the total daily insulin dose declined significantly (median: 1.10-0.70 units/kg/day). While intensified monitoring might increase insulin dosing, our data suggest improved efficiency, potentially due to reduced hyperglycemia, optimized basal-bolus regimens, and healthier behaviors prompted by CGM feedback [27]. Although we did not directly measure behavioral changes, pediatric literature supports that diabetes technology fosters better self-management [27]. Crucially, insulin dose reduction occurred without weight gain, alleviating concerns about adverse metabolic effects from intensive therapy [28], consistent with prior findings that technology-assisted glycemic improvement does not necessarily raise insulin needs [29,30]. Future research should examine whether CGM use promotes lifestyle changes, enhancing insulin sensitivity.

Patient-reported outcomes revealed high satisfaction (CGM-SAT 4.8/5; benefits of CGM 4.9/5 and hassles of CGM 4.6/5), reflecting positive experiences with the Linx™ device. Adolescents strongly agreed that CGM improved their diabetes management and quality of life, outweighing inconveniences. This contrasts with earlier evidence reporting mixed psychosocial effects due to device burdens like alarms and visibility, which sometimes caused annoyance or family conflict [31]. Our findings align with recent data showing newer-generation CGM systems with fewer hassles are better received [20,25,26]. For example, the CITY trial extension reported increased satisfaction after 12 months using Dexcom G6 [20], and a 2023 study linked higher CGM satisfaction with improved %TIR70-180, reduced glycemic risk, and better quality of life regardless of insulin delivery method [25]. The coexistence of high satisfaction, excellent adherence, and improved glycemia supports the idea that when adolescents find CGM acceptable and valuable, consistent use and clinical benefit follow. Barriers such as cost, discomfort, alarm fatigue, and stigma have limited CGM uptake historically [20]. Still, the Linx’s factory calibration, extended wear, and free provision likely mitigated these issues in our study. This, combined with advances in CGM technology and enhanced support, bodes well for wider, sustained CGM adoption, as satisfaction drives long-term adherence [25].

The clinical implications are substantial: integrating CGM into care for adolescents with suboptimal control can yield rapid, meaningful improvements in both biomedical and psychosocial outcomes. Lowering HbA1c during adolescence is critical to reducing the risk of long-term microvascular and cardiovascular complications [24]. Increased %TIR70-180 translates to more hours per day spent in healthy glucose ranges, likely improving energy and daily well-being. At the same time, the sharp decrease in hypoglycemic events reduces acute risks and alleviates fear and anxiety associated with lows. The high satisfaction and willingness to continue CGM use indicate that modern devices like Linx™ address many previous barriers to adolescent uptake and adherence [20]. Clinicians should emphasize comprehensive education and follow-up to maximize device adoption and long-term success. From a public health perspective, CGM is a cost-effective intervention that can reduce emergency care visits and costly complications, particularly relevant in resource-limited settings where CGM access remains low. Our findings support expanding CGM coverage and support services to improve diabetes outcomes at a population level.

This study has some limitations to consider. First, as an open-label, single-arm investigation without a concurrent control group, it is not applicable to attribute the observed improvements in glycemic control solely to CGM use. While the magnitude of HbA1c reduction observed over three months exceeds typical trends in adolescent diabetes care, where control often remains stable or worsens, randomized controlled trials are needed to definitively establish causality. Second, the relatively short follow-up period limits insight into the long-term sustainability of benefits and adherence. However, continued improvements during the study and supportive real-world data suggest potential durability. Third, the single-center design and modest sample size, with participants who volunteered and were motivated to adopt CGM, may limit generalizability to broader or less engaged populations. Fourth, we did not systematically assess psychosocial outcomes such as diabetes distress or quality of life, nor minor device-related issues like alarm fatigue or skin irritation; however, no participants discontinued use due to such concerns. Finally, cost and access remain significant barriers to widespread CGM adoption, particularly in resource-limited settings. The free provision of CGM and dedicated support in this study likely facilitated high adherence and favorable outcomes, conditions that may not be universally achievable. Thus, ensuring access to CGM alongside comprehensive education and ongoing support will be essential to fully realize its benefits in routine clinical care.

Future research should include randomized controlled trials of Linx™ and similar next-generation CGM devices in adolescents, with longer follow-up and comprehensive patient-reported outcomes such as quality of life and diabetes distress. Integrating hybrid closed-loop insulin delivery systems warrants investigation, as combined technology may further improve outcomes. Head-to-head comparisons of CGM systems and qualitative research on barriers like alarm fatigue and social stigma would inform personalized device choice and support strategies. Behavioral interventions combined with CGM use may enhance engagement and glycemic outcomes. Exploring satisfaction as a mediator of adherence and metabolic control could optimize clinical approaches. Additionally, studies on earlier CGM initiation before adolescence and cost-effectiveness analyses in diverse healthcare settings would support prevention efforts and inform policy decisions.

## Conclusions

In conclusion, using a 15-day factory-calibrated CGM (Linx™) for three months led to clinically significant improvements in glycemic control and high user satisfaction in adolescents with poorly controlled T1D. We observed a substantial reduction in HbA1c alongside increased %TIR70-180 and reduced hypoglycemia, indicating better overall glucose management without added risk. Participants’ high satisfaction with CGM suggests that modern sensor technology is well-accepted by teens, potentially facilitating sustained adherence and continued glycemic benefit. These findings reinforce the role of CGM as a powerful tool to address the unique challenges of diabetes management in adolescence, bridging the gap between suboptimal real-world control and recommended targets. Widespread implementation of CGM in youth, coupled with appropriate support, could substantially improve short- and long-term health outcomes in this vulnerable population. Future studies should build on these results to help translate CGM’s proven efficacy into routine global practice, enabling more youth with T1D to achieve safer glucose control, better quality of life, and improved long-term health.
